# Draft Genome Sequence of Enterobacter asburiae UFMG-H9, Isolated from Urine from a Healthy Bovine Heifer (Gyr Breed)

**DOI:** 10.1128/MRA.00385-20

**Published:** 2020-05-21

**Authors:** Silvia Giannattasio-Ferraz, Laura Maskeri, André Penido Oliveira, Edel F. Barbosa-Stancioli, Catherine Putonti

**Affiliations:** a Departamento de Microbiologia, Instituto de Ciências Biológicas, Universidade Federal Minas Gerais, Belo Horizonte, MG, Brazil; b Bioinformatics Program, Loyola University Chicago, Chicago, Illinois, USA; c Empresa de Pesquisa Agropecuária de Minas Gerais, Uberaba, MG, Brazil; d Department of Biology, Loyola University Chicago, Chicago, Illinois, USA; e Department of Computer Science, Loyola University Chicago, Chicago, Illinois, USA; f Department of Microbiology and Immunology, Stritch School of Medicine, Loyola University Chicago, Maywood, Illinois, USA; Broad Institute

## Abstract

Enterobacter asburiae is part of the Enterobacter cloacae complex, related to nosocomial opportunistic infections in humans. Here, we report the draft genome of E. asburiae strain UFMG-H9, an isolate from urine from a healthy Gyr heifer.

## ANNOUNCEMENT

Enterobacter asburiae is part of the Enterobacter cloacae complex, which includes six species of the genus. Species of this complex are widely distributed and known as commensal species and opportunistic pathogens with the potential to cause nosocomial infections in humans (1). E. asburiae has also been identified as an antibiotic-resistant species (2), and although it has not been reported in livestock animals so far, it was recently reported in fruits and vegetables (3), increasing concerns about the dissemination of this pathogen. Here, we announce the draft genome sequence of E. asburiae strain UFMG-H9. This strain was isolated from a healthy heifer in Brazil. The animal belongs to a pure-by-origin Gyr herd, from the Agricultural Research Company of Minas Gerais state.

The present study was approved by the Ethics Committee in Animal Experimentation of the Universidade Federal de Minas Gerais, Brazil (approval number 40/2019), and samples were collected in May 2019, with all of the experiments being performed in accordance with relevant guidelines. For sampling, the vulva of the animal was washed with soap and distilled water, and then midstream urine collection was made using a 50-ml sterile tube. The material was frozen and kept at −20°C until processing (within 48 h). Two-milliliter aliquots were centrifuged, and the supernatant was plated onto lysogeny broth (LB) agar plates. The plates were incubated overnight at 37°C. Single colonies were selected, grown in LB cultures overnight at 37°C, and plated again. In order to obtain pure colonies, this process was repeated at least three times. A single colony was then inoculated in LB liquid medium and grown overnight at 37°C with agitation. A 1-ml aliquot was centrifuged, and the pellet was used for DNA extraction using the DNeasy UltraClean microbial kit (Qiagen, Hilden, Germany). The genus and species were determined by sequencing of the 16S rRNA gene using the 63f and 1387r primers (4). The amplicons were sequenced by Genewiz (South Plainfield, NJ, USA) via Sanger sequencing, and the sequence produced was queried against the NCBI 16S rRNA sequence database. DNA was quantified with a Qubit fluorometer and sent to the Microbial Genomic Sequencing Center at the University of Pittsburgh for whole-genome sequencing. To prepare the library, the DNA was fragmented using an Illumina tagmentation enzyme, and indices were attached using PCR. The genome was sequenced using the NextSeq 550 platform. Sequencing produced 1,406,751 pairs of 150-bp reads, which were trimmed using Sickle v1.33 (https://github.com/najoshi/sickle). Once trimmed, the genome was assembled using SPAdes v3.13.0 with the only-assembler option for k values of 55, 77, 99, and 127 (5). The genome coverage was then calculated using BBMap v38.47 (https://sourceforge.net/projects/bbmap). After that, the genome was annotated by the NCBI Prokaryotic Genome Annotation Pipeline (PGAP) v4.11 (6). Unless noted otherwise, default parameters were used for all software.

The E. asburiae UFMG-H9 draft genome is 5,039,202 bp long with a GC content of 52.71%. The assembly includes 116 contigs with an *N*_50_ value of 139,398 bp, and the genome coverage was 77×. Annotation identified 4,765 coding genes and 77 tRNAs. The genome sequence was examined using ResFinder v3.2 (7), which revealed several antibiotic resistances (Table 1). Future analysis regarding the pathogenic potential of this strain would further our knowledge of E. asburiae circulation.

**TABLE 1:** Predicted antibiotic resistances for E. asburiae UFMG-H9

Resistance gene	Predicted phenotype
*bla*_ACT-6_	β-Lactam resistance (AmpC type)
*fosA*	Fosfomycin resistance
*qnrB2*	Quinolone resistance
*sul1*	Sulfonamide resistance
*dfrA25*	Trimethoprim resistance

### Data availability.

This whole-genome sequencing project is available in GenBank under the accession number JAAVSG000000000. Raw reads were deposited in the SRA under accession number SRR11455637. This sequencing effort is part of BioProject accession number PRJNA615899.
